# Impact of dietary inclusion of artificial saliva as buffer on rumen physico-chemistry, trace mineral dynamics and metabolic alterations in lambs

**DOI:** 10.3389/fvets.2026.1854038

**Published:** 2026-06-03

**Authors:** Mutassim M. Abdelrahman, Ibrahim A. Alhidary, Mohammed M. Qaid, Mohsen M. Alobre, Abdulkareem M. Matar, Ramzi A. Amran, Riyadh S. Aljumaah

**Affiliations:** 1 Department of Animal Production, College of Food and Agricultural Sciences, King Saud University, Riyadh, Saudi Arabia; 2 Department of Zoology, College of Science, King Saud University, Riyadh, Saudi Arabia

**Keywords:** artificial saliva, lambs, metabolic alterations, rumen physico-chemistry, trace mineral status

## Abstract

This study evaluated the effects of supplementing a total mixed ration (TMR) with graded levels of artificial saliva (AS) on rumen fermentation, trace mineral status, metabolic responses, and carcass traits of growing lambs using a completely randomized design. Forty-five lambs were assigned to diets containing 0, 1.5, 3.0, 4.5, or 6% AS. Artificial saliva supplementation produced clear dose-dependent responses. Moderate AS levels (1.5–4.5%) improved ruminal pH stability (*p* = 0.002) and increased acetic and propionic acid production (*p* < 0.01), supporting improved rumen function. Ruminal fluid Cu, Zn, and Se concentrations increased with AS level (*p* < 0.0001), particularly at 6%, indicating enhanced mineral solubility. However, excessive AS (6%) reduced VFA production (*p* < 0.05), increased NEFA (*p* < 0.01), decreased serum glucose (*p* = 0.0004), and reduced visceral and adipose tissue weights (*p* < 0.05), suggesting metabolic imbalance. Meat initial and ultimate pH were also higher at 6% AS (*p* < 0.0001), reflecting reduced glycogen reserves. Correlation analysis revealed strong associations between trace minerals (Cu, Zn, Se) and key physiological indicators, including NEFA, meat pH, and rumen color attributes, highlighting the integrative relationship between mineral metabolism, rumen function, and whole-body energy status. In conclusion, moderate AS inclusion improved rumen fermentation and maintained metabolic stability.

## Highlights

- Moderate artificial saliva (AS) supplementation (1.5%–4.5%) optimized ruminal pH, enhanced VFA production, and supported normal rumen epithelial development in growing lambs.
- Excessive AS inclusion (6%) impaired rumen fermentation, reduced VFA concentrations, altered rumen papillae development, and increased metabolic stress indicators (elevated NEFA and reduced glucose).
- Moderate AS levels improved trace mineral status, particularly rumen Cu, Zn, and Se, without affecting Mn, Fe, or Co.
- High AS inclusion increased meat ultimate pH and reduced visceral and adipose tissue reserves, indicating compromised pre-slaughter energy metabolism and carcass quality.

## 1 Introduction

Ruminants require a stable ruminal environment to maintain efficient fermentation and volatile fatty acid (VFA) production, which are essential for energy supply and nutrient utilization (1–4). Under high-concentrate feeding systems, rapid fermentation may lower ruminal pH and impair microbial activity and epithelial integrity (5–7). Saliva plays a major role in maintaining ruminal homeostasis through its bicarbonate- and phosphate-based buffering capacity (8–11). Therefore, dietary buffering agents such as sodium bicarbonate and phosphate salts have been widely used to stabilize ruminal pH, although their effects on fermentation and animal performance have been inconsistent (12–16). Artificial saliva (AS), formulated to mimic the buffering and ionic properties of natural saliva, may provide broader physiological effects than conventional buffers (17, 18). However, limited information is available regarding its influence on ruminal fermentation, trace mineral status, metabolic responses, and carcass characteristics in growing lambs.

Trace minerals such as Cu, Zn, Se, Fe, Mn, and Co are essential for antioxidant defense, immune function, epithelial maintenance, and energy metabolism in ruminants (19, 20). Their availability and solubility are influenced by ruminal conditions including pH and ionic balance (21, 22). Alterations in ruminal physicochemistry may therefore affect mineral dissociation and utilization, with potential consequences for systemic metabolism and tissue development (23). Because saliva naturally contributes minerals and buffering compounds to the rumen, supplementation with AS may influence these processes through modifications in the ruminal environment.

Although previous studies have evaluated conventional buffering agents (24–27), information remains limited regarding the effects of multi-ionic artificial saliva supplementation on ruminal trace mineral concentrations and associated metabolic responses in lambs. Furthermore, few studies have examined the relationships among ruminal fermentation, mineral dynamics, energy metabolism, visceral tissue development, and carcass characteristics under different buffering conditions (27). Addressing these relationships may improve understanding of how ruminal buffering strategies influence animal physiology and productive responses.

The novelty of the present study lies in evaluating AS not only as a buffer but also as a mineral and ionic modulator capable of altering trace mineral homeostasis and translating those changes into measurable effects on metabolism, visceral tissue development, and meat quality. We hypothesized that moderate AS supplementation would improve ruminal fermentation and metabolic stability, whereas excessive inclusion could adversely affect ruminal function and associated physiological responses. Accordingly, this study aimed to evaluate the effects of graded levels of AS supplementation on ruminal pH, VFA production, ruminal trace mineral concentrations, metabolic indicators, visceral organ development, epithelial morphology, and carcass characteristics in growing lambs fed a total mixed ration.

## 2 Materials and methods

### 2.1 Ethical approval

All procedures involving animals-including husbandry, sampling, and slaughter-were approved by the Scientific Ethics Committee of King Saud University, Saudi Arabia (KSU-SE-23-70), and performed in accordance with institutional and national welfare guidelines.

### 2.2 Animals, housing, and experimental design

Two weeks after weaning (75–85 days of age), 45 growing male Naemi lambs were enrolled in an 84-day feeding trial. Following a 2-week adaptation period, pens were randomly assigned to five dietary treatments in a completely randomized design. Each treatment comprised three pens with three lambs per pen (nine lambs per treatment), with an initial average body weight of 23 ± 1.8 kg. The animals were housed in 15 pens of 1.2 × 150 m^2^. Pens, each equipped with a feeder and waterer, served as the experimental units.

Animals were *fed ad libitum* for 84 days using five isonitrogenous and isoenergetic total mixed rations (TMR). Diets consisted of a TMR supplemented with graded levels of artificial saliva (AS): 0, 1.5, 3.0, 4.5, or 6% AS/TMR diet (providing approximately 0, 22.5, 45, 67.5, and 90 g/lamb/day, respectively). The inclusion levels of AS were selected based on our previous study Abdelrahman, Ibrahim A. Alhidary (11) on growth performance indices and have been applied consistently in the present experiment. The AS product was supplied as a powdered mixture containing sodium bicarbonate, dipotassium phosphate, monoammonium phosphate, and magnesium sulfate (manufactured by David Taylor Animal Nutrition Ltd., Carrick Mill, Collinstown, Co. Westmeath, Ireland), formulated to simulate the ionic and buffering properties of natural ruminant saliva. The analyzed mineral composition consisted of 0.01% calcium, 17.19% sodium, 8.24% phosphorus, and 0.09% magnesium. The TMR consisted of barley grain (30.50%), wheat feed (26.00%), wheat bran (5.00%), palm kernel cake (17.50%), soybean hulls (13.40%), salt (1.00%), limestone (2.50%), molasses (3.00%), an acid buffer (1.00%), and a commercial premix (0.10%).

### 2.3 Serum sampling and biochemical analyses

Blood samples (5 mL) were collected via jugular venipuncture from all lambs on days 1, 42, and 84. Samples were centrifuged at 2,400×g for 15 min at 4 °C to obtain serum. Glucose and non-esterified fatty acids (NEFA) were measured using commercial kits (Randox Laboratories, Antrim, United Kingdom) and a microplate reader (Multiskan EX, Thermo Fisher Scientific, Waltham, MA, United States).

### 2.4 Rumen sampling and minerals analysis

Ruminal fluid samples were collected from the same animals at d 84 of experiment using a stomach tube attached to a gentle vacuum pump following standard veterinary procedures. Each treatment consisted of one sample collected from each pen, resulting in five samples per treatment. Trace mineral concentrations (Cu, Zn, Se, Fe, Mn, Co) in rumen fluid were collected from selected lambs in each treatment group using an oral stomach tube connected to a vacuum pump. To minimize saliva contamination, the initial 50 mL was discarded following Abdelrahman, Swelum (28). Subsequently, 50 mL of rumen fluid was collected from each lamb at the end of the experimental period.

Samples were immediately transferred into labeled glass tubes and filtered through four layers of cotton gauze. The filtrate was centrifuged at 1,000×g for 10 min, after which the supernatant was carefully transferred into labeled Eppendorf tubes and stored at −20 °C until analysis. Micro-mineral concentrations were determined using ICP-OES equipped with a MEINHARD Type A2 nebulizer, as described by Abdelrahman, Swelum (28).

### 2.5 Slaughter procedures and carcass measurements

At the end of the trial (day 84), 20 lambs (four lambs per treatment) were randomly selected, fasted for 16 h. Animals were slaughtered following the Halal method. The longissimus dorsi muscle was used for physicochemical analyses. Initial and ultimate meat pH (15 min and 24 h postmortem) and initial core temperature were measured using a digital pH meter equipped with a puncture electrode and thermometer (Hanna Instruments, RI, United States) (29).

Carcass measurements included backfat thickness, body wall fat thickness, omental fat mass, and absolute weights of full and empty stomach and intestines. The 6th–13th ribs of right side of each carcass were separated. Fat depot components, including backfat thickness over the 6th and 10th ribs and body wall fat thickness, were measured using a digital caliper, while omental fat was weighed using an electronic balance.

### 2.6 Rumen fermentation profile and tissue color measurements

Rumen fluid (~50 mL) from four lambs per treatment was collected immediately after slaughter, acidified with 2 mL of concentrated H₂SO₄, and stored at −20 °C. Thawed samples were analyzed for acetate and propionate using a gas chromatograph equipped with a capillary column (Agilent Technologies, Wilmington, DE) and 2-ethylbutyric acid as an internal standard, following Alharthi, Alshamiry (30).

Rumen pH was measured 30 min postmortem using a digital pH-meter (Model pH 211, Hanna Instruments) after straining through cheesecloth.

Tissue samples from the rumen, reticulum, and omasum (four samples per treatment) were collected for color analysis. Color values (L*, a*, b*) were determined using a Minolta Chroma Meter (CR-400, Konica Minolta, Japan) based on the CIELAB system.

### 2.7 Statistical analysis

All data were analyzed using one-way ANOVA in a completely randomized design (PROC GLM; SAS 9.4, SAS Institute Inc., Cary, NC). The statistical model used was:

Yij=μ+Ti+eij

where $Y_{\mathrm{ij}}$ is the observation for treatment i and animal j ; μ is the overall mean, $T_{i}$ is the fixed effect of treatment, and $e_{\mathrm{ij}}$ is the residual error. Mean differences among treatments were assessed using the Ryan–Einot–Gabriel–Welsch multiple range test. Because serum glucose and NEFA concentrations were evaluated at discrete sampling points (days 42 and 84), the statistical analysis focused primarily on treatment effects within each sampling period and on overall treatment means rather than on longitudinal temporal responses. Statistical significance was declared at *p* < 0.05.

## 3 Results

The calculated chemical composition of the TMR contained 89.5% dry matter (DM). On a DM basis, it comprised 13.30% crude protein, 12.95% crude fiber, and 2.50% ether extract (EE), providing a metabolizable energy value of 2.79 Mcal/kg.

### 3.1 Ruminal fluid trace mineral profile

Dietary inclusion of artificial saliva (AS) in the TMR significantly influenced several ruminal fluid trace minerals (Table 1). Ruminal fluid Cu, Zn, and Se concentrations exhibited marked treatment effects (*p* < 0.0001). Lambs receiving 6% AS showed substantially higher Cu (1.45 mg/L) and Zn (0.994 mg/L) compared with all other groups, demonstrating strong linear responses (*p* < 0.0001). Selenium followed a similar pattern, with the 1.5 and 6% AS groups showing the greatest concentrations (151.7 and 154.0 μg/L, *p* < 0.0001). In contrast, Mn, Fe, and Co were not significantly affected by treatment (*p* > 0.25).

**Table 1:** Effects of feeding a total mixed ration (TMR) containing varying levels of artificial saliva on rumen trace mineral levels, in growing lambs.

Parameters	Artificial saliva (%)					Standard error	*p* value	Contrast			
	0	1.5	3	4.5	9			Linear	Quadratic	Cubic	Quartic
Mn, μg/L	4.17	4.11	4.00	3.77	4.13	0.178	0.321	0.913	0.137	0.170	0.462
Fe, mg/L	1.35	1.43	1.46	1.35	1.45	0.060	0.253	0.268	0.869	0.104	0.222
Co, μg/L	0.489	0.499	0.479	0.481	0.453	0.019	0.865	0.432	0.916	0.456	0.975
Cu, mg/L	0.878^b^	1.01^b^	0.959^b^	1.00^b^	1.45^a^	0.040	<0.0001	<0.0001	0.0003	0.001	0.931
Zn, mg/L	0.635^d^	0.774^b^	0.721^bc^	0.669^cd^	0.994^a^	0.019	<0.0001	<0.0001	0.0001	<0.0001	0.390
Se, μg/L	114.0^c^	151.7^a^	121.0^bc^	133.0^ab^	154.0^a^	4.94	<0.0001	0.001	0.689	0.0003	0.002

Values within a row bearing different lowercase superscript letters differ significantly at *p* < 0.05.

### 3.2 Serum NEFA and glucose dynamics

The mobilization of body energy reserves, assessed through NEFA and glucose, responded markedly to AS supplementation (Table 2). NEFA concentrations at day 42 increased sharply in lambs fed 3 and 6% AS (5.04 and 7.08 mg/dL; *p* < 0.0001), displaying a strong positive linear trend. By day 84, NEFA remained elevated in the 1.5% group (4.32 mg/dL), although the highest treatment (6% AS) did not differ from the control. Average NEFA across the trial was greatest at 6% AS (5.10 mg/dL; *p* < 0.0001).

**Table 2:** Effects of feeding a total mixed ration (TMR) with increasing levels of artificial saliva on serum glucose (mg/dL) and NEFA (mg/dL) concentrations at mid-experiment (day 42), end-experiment, and overall, as an indicator of energy mobilization in growing lambs.

Parameters	Artificial saliva (%)					Standard error	*P* value	Contrast			
	0	1.5	3	4.5	9			Linear	Quadratic	Cubic	Quartic
NEFA at day 42	2.16^c^	2.58^c^	5.04^b^	2.70^c^	7.08^a^	0.370	<0.0001	<0.0001	0.815	0.215	0.617
NEFA at day 84	2.79^b^	4.32^a^	2.76^b^	2.64^b^	3.12^b^	0.168	<0.0001	0.030	0.225	0.184	0.704
Average NEFA	2.48^d^	3.45^bc^	3.90^b^	2.67^cd^	5.10^a^	0.240	<0.0001	<0.0001	0.314	0.079	0.924
Glucose at day 42	60.8^a^	45.4^ab^	43.1^b^	36.1^bc^	24.9^c^	4.22	0.0004	<0.0001	0.815	0.215	0.617
Glucose at day 84	53.8	42.1	39.3	43.1	37.8	4.09	0.990	0.030	0.225	0.184	0.704
Average glucose	57.3^a^	43.7^b^	41.2^bc^	39.6^bc^	31.3^c^	2.96	0.0004	<0.0001	0.314	0.079	0.924

Values within a row bearing different lowercase superscript letters differ significantly at *p* < 0.05. NEFA, non-esterified fatty acids. At the start of the experiment, the baseline mean (data at time 0) serum glucose concentration was 53.6 mg/dL, while NEFA averaged 2.52 mg/dL.

Conversely, glucose concentrations decreased progressively with higher AS levels. Glucose at day 42 declined from 60.8 mg/dL in the control to 24.9 mg/dL at 6% AS (*p* = 0.0004). A similar pattern was observed for mean glucose, with a clear linear reduction across treatments (*p* < 0.0001), indicating increased energy mobilization as an AS inclusion increased.

### 3.3 Postmortem muscle pH and internal organ components

Artificial saliva exerted significant effects on meat acidity and visceral components (Table 3). Both initial and ultimate pH values increased at the highest AS level (6%), with initial pH rising to 6.15 and ultimate pH to 5.97 (*p* < 0.0001), following strong linear and cubic trends.

**Table 3:** Effects of feeding a total mixed ration (TMR) with varying levels of artificial saliva on postmortem meat characteristics (initial and ultimate pH, core temperature) and on internal components of the digestive organs and fat in growing lambs.

Parameters	Artificial saliva (%)					Standard error	*P* value	Contrast			
	0	1.5	3	4.5	9			Linear	Quadratic	Cubic	Quartic
Initial pH	5.82^b^	5.84^b^	5.88^b^	5.81^b^	6.15^a^	0.025	<0.0001	<0.0001	<0.0001	0.000	0.008
Initial core temperature	25.75	25.10	25.45	25.50	24.85	0.077	0.077	<0.0001	0.314	<0.0001	0.182
Ultimate pH	5.71^b^	5.74^b^	5.69^b^	5.72^b^	5.97^a^	0.021	<0.0001	<0.0001	<0.0001	0.000	0.954
Component weight											
Back fat (mm)	11.67^b^	9.90^b^	17.51^a^	12.86^b^	2.49^c^	0.744	<0.0001	<0.0001	<0.0001	<0.0001	0.000
Body fat (mm)	10.11^b^	8.13^b^	12.55^a^	10.29^b^	3.00^c^	0.597	<0.0001	<0.0001	<0.0001	<0.0001	0.010
Stomach full (kg)	4.56^a^	4.41^a^	4.79^a^	4.09^a^	2.88^b^	0.278	0.002	0.001	0.008	0.257	0.368
Stomach empty (kg)	1.55^a^	1.50^a^	1.58^a^	1.45^a^	0.95^b^	0.078	0.0002	0.0001	0.002	0.062	0.845
Intestine full (kg)	2.75^a^	2.50^a^	2.45^ab^	2.45^ab^	2.06^b^	0.101	0.004	0.0004	0.559	0.085	0.737
Omental fat (kg)	0.57^ab^	0.63^a^	0.46^ab^	0.73^a^	0.22^b^	0.092	0.014	0.065	0.057	0.074	0.028

Values within a row bearing different lowercase superscript letters differ significantly at *p* < 0.05.

Internal fat depots were highly responsive. Back fat and body fat increased sharply at 3% AS (17.51 and 12.55 mm, respectively) but were lowest at 6% AS (2.49 and 3.00 mm; *p* < 0.0001). Similarly, stomach full and empty weights, intestine weight, and omental fat all declined markedly in the 6% AS group (*p* ranging from 0.0002 to 0.014), implying reductions in gut fill and adipose reserves.

### 3.4 Ruminal fermentation profile

Ruminal fluid pH, acetic acid, and propionic acid were significantly affected by AS level (Table 4). Ruminal pH at 1 h postmortem increased significantly (*p* = 0.002) at 1.5 and 3% AS, reaching the highest value in the 3% group (5.94), whereas lambs receiving 0% AS recorded the lowest pH (5.18). Ruminal acetic acid concentrations were also influenced by AS level (*p* = 0.002), with the 3 and 4.5% groups exhibiting the highest acetate concentrations (33.47 and 32.63 mmol/L, respectively), both significantly greater than the 0, 1.5, and 6% groups. Propionic acid showed a similar pattern (*p* < 0.0001), peaking at 3% (31.78 mmol/L) and 4.5% (29.35 mmol/L), while remaining significantly lower in the control, 1.5, and 6% groups. Consequently, the acetate: propionate ratio declined sharply at moderate AS levels (*p* = 0.0021), with the lowest ratios observed at 3% (1.06) and 4.5% (1.13), compared with the highest ratio in the control group (2.06). Notably, the 6% AS group exhibited an intermediate ratio (1.51), reflecting reduced propionate output relative to the moderate-AS treatments. Collectively, these findings indicate that moderate AS supplementation (3–4.5%) optimizes ruminal pH, enhances fermentation toward greater propionate production, and shifts the acetate: propionate balance toward more efficient energy metabolism, whereas excessive AS (6%) partially disrupts these improvements.

**Table 4:** Effects of feeding a total mixed ration (TMR) containing varying levels of artificial saliva (AS) on ruminal acetic and propionic acid concentrations, as well as average pH at 1 h PM, in growing lamb.

Parameters	Artificial saliva (%)					SE	*p* value	Contrast			
	0	1.5	3	4.5	9			Linear	Quadratic	Cubic	Quartic
Ruminal fluid											
Average pH at 1 h PM	5.18^b^	5.70^a^	5.94^a^	5.53^ab^	5.52^ab^	0.105	0.002	0.140	0.001	0.064	0.130
Acetic acid (mmL)	26.54^ab^	19.87^b^	33.47^a^	32.63^a^	21.16^b^	2.289	0.002	0.786	0.013	0.001	0.063
Propionic acid (mmL)	13.04^b^	16.12^b^	31.78^a^	29.35^a^	14.27^b^	2.005	<0.0001	0.026	<0.0001	0.001	0.048
Acetate: propionate ratio	2.06^a^	1.26^b^	1.06^b^	1.13^b^	1.51^b^	0.152	0.0021	0.022	0.0003	0.552	0.773

Values within a row bearing different lowercase superscript letters differ significantly at *p* < 0.05.

### 3.5 Digestive organ color attributes

Colorimetric characteristics of rumen, reticulum, and omasum tissues responded to AS inclusion (Table 5). Rumen lightness (L^*^) increased markedly at 6% AS (42.9; *p* < 0.0001), whereas redness (a^*^) and yellowness (b^*^) were not significantly altered. Reticulum yellowness (b^*^) increased only at 3% AS (*p* = 0.006). In the omasum, redness (a^*^) was highest at 3% AS (22.1; *p* = 0.009), reflecting diet-induced mucosal pigmentation changes.

**Table 5:** Effects of feeding a total mixed ration (TMR) with increasing levels of artificial saliva on the color of digestive organs in growing lambs.

Parameters	Artificial saliva (%)					Standard error	*P* value	Contrast			
	0	1.5	3	4.5	9			Linear	Quadratic	Cubic	Quartic
Rumen color											
L^i*^	38.6^b^	34.9^c^	34.7^c^	39.8^ab^	42.9^a^	0.965	<0.0001	0.001	<0.0001	0.088	0.266
a^i*^	3.23	2.70	2.48	3.12	3.40	0.222	0.053	0.296	0.009	0.364	0.354
b^i*^	12.3	10.2	9.5	11.5	11.6	0.680	0.066	0.992	0.014	0.150	0.318
Reticulum color											
L^i*^	32.2	31.8	32.4	29.2	34.4	1.245	0.113	0.665	0.128	0.081	0.123
a^i*^	15.1	16.0	16.0	15.9	16.5	0.327	0.103	0.018	0.661	0.175	0.990
b^i*^	2.33^b^	2.27^b^	3.14^a^	2.55^b^	2.48^b^	0.148	0.006	0.231	0.018	0.402	0.003
Omasum color											
L^i*^	38.5	37.6	38.4	39.9	38.7	0.915	0.527	0.335	0.990	0.154	0.800
a^i*^	19.4^b^	21.4^ab^	22.1^a^	19.7^b^	19.9^b^	0.531	0.009	0.658	0.004	0.037	0.133
b^i*^	7.87	8.16	7.09	6.51	7.99	0.555	0.231	0.432	0.187	0.072	0.951

Values within a row bearing different lowercase superscript letters differ significantly at *p* < 0.05. L*, a*, and b* refer to lightness, redness, and yellowness.

### 3.6 Correlation analysis

Pearson correlations (Table 6) revealed strong physiological linkages across metabolic, carcass, and ruminal variables. NEFA was negatively correlated with glucose (*r* = −0.57; *p* < 0.01) and with fat depots, stomach weight, and intestine weight (*r* = −0.58 to −0.67; *p* < 0.05), confirming that higher NEFA was associated with reduced body and visceral reserves. Muscle ultimate pH was positively correlated with NEFA (*r* = 0.71; *p* < 0.01) and with trace minerals Cu, Zn, and Se (*r* = 0.60–0.90; *p* < 0.01).

**Table 6:** Pearson’s correlation coefficients among parameters of growing lambs fed a total mixed ration supplemented with artificial saliva.

Pearson correlation coefficients, *N = 20 “Prob > |r*| under H0: Rho = 0”																				
	NEFA	GLU	Meat pHu	Back fat	Body fat	Stomach	Intestine	Omental	Rumen trace mineral						Rumen			Ruminal fluid		
						wt.	wt.	fat	Mn	Fe	Co	Cu	Zn	Se	L*	a*	b*	pH	Acetic	Propionic
NEFA	1																			
Glucose	−0.57^**^	1																		
Meat pHu	0.71^**^	−0.62^**^	1																	
Back fat	−0.51^*^	0.3	−0.82^**^	1																
Body fat	−0.64^*^	0.37	−0.87^**^	0.96^**^	1															
Stomach wt.	−0.64^*^	0.43	−0.75^**^	0.81^**^	0.83^**^	1														
Intestine wt.	−0.67^**^	0.57^*^	−0.58^**^	0.50^*^	0.56^**^	0.75^**^	1													
Omental fat	−0.58^*^	0.26	−0.47^*^	0.42	0.44^*^	0.65^**^	0.58^*^	1												
Mn	0.39^*^	0.19	0.22	−0.48^*^	−0.48^*^	−0.45	−0.18	−0.49^*^	1											
Fe	0.45^*^	−0.21	0.21	−0.14	−0.14	−0.22	−0.50^*^	−0.39	0.19	1										
Co	0.06	0.42	−0.04	−0.15	−0.11	−0.19	−0.15	−0.21	0.4	0.47	1									
Cu	0.79^**^	−0.58^**^	0.90^**^	−0.81^**^	−0.87^**^	−0.85^**^	−0.73^**^	−0.49^*^	0.28	0.2	0.07	1								
Zn	0.77^**^	−0.67^**^	0.83^**^	−0.78^**^	−0.78^**^	−0.76^**^	−0.73^**^	−0.61^*^	0.31	0.51^*^	0.05	0.84^**^	1							
Se	0.49^*^	−0.49	0.60^**^	−0.68^*^	−0.72^**^	−0.57^*^	−0.49^*^	−0.2	0.39	0.28	0.19	0.61^**^	0.69^**^	1						
L* rumen	0.22	−0.27	0.63^**^	−0.62^**^	−0.62^**^	−0.73^**^	−0.41	−0.34	0.17	0.1	0.04	0.55^*^	0.44	0.33	1					
a* rumen	0.12	−0.14	0.42	−0.47^*^	−0.48^*^	−0.27	0.05	−0.02	0.29	−0.47^*^	−0.51^*^	0.33	0.14	0.14	0.42	1				
b* rumen	−0.15	0.27	0.17	−0.4	−0.33	−0.24	0.12	−0.04	0.37	−0.27	−0.07	0.14	0	0.02	0.38	0.50^*^	1			
Rumen pH	0.29	−0.44	−0.12	0.33	0.21	0.09	−0.34	−0.09	−0.07	0.41	0.03	−0.02	0.16	0.25	−0.28	−0.51	−0.64^*^	1		
Acetic acid	−0.29	0.06	−0.43	0.61^*^	0.60^**^	0.36	0.08	0.39	−0.4	0	−0.02	−0.32	−0.46	−0.45	−0.16	−0.36	−0.17	0.29	1	
Propionic acid	−0.16	−0.27	−0.42	0.65^*^	0.60^**^	0.3	0	0.32	−0.44	−0.02	−0.19	−0.26	−0.29	−0.22	−0.23	−0.36	−0.47^*^	0.61^**^	0.74^**^	1
Labeling	1	0.9	0.8	0.7	0.6	0.5	0.4	0.3	0.2	0.1	−0.1	−0.2	−0.3	−0.4	−0.5	−0.6	−0.7	−0.8	−0.9	−1

*<0.05, **<0.01 NEFA: non-esterified fatty acids.

Ruminal color parameters (especially L^*^) were significantly associated with rumen pH and trace mineral status. Notably, propionic acid showed a strong positive correlation with acetic acid (*r* = 0.74; *p* < 0.01) and rumen pH (*r* = 0.61; *p* < 0.01), indicating synchronized fermentation responses.

### 3.7 Rumen morphology

**Figure 1:**
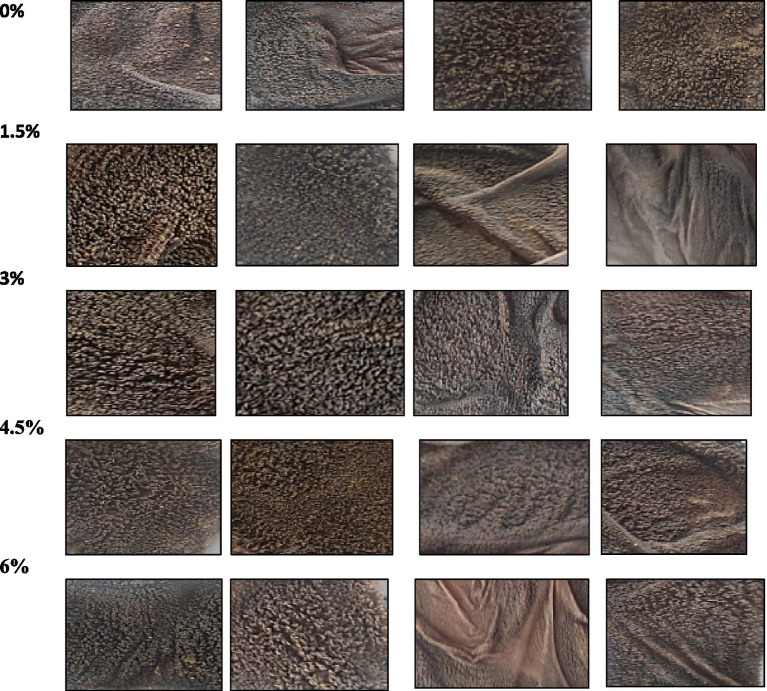
Effects of feeding a total mixed ration (TMR) with increasing levels of artificial saliva on rumen color and papillae morphology in growing lambs.

Visual evaluation of rumen epithelial morphology (Figure 1) confirmed the quantitative rumen color results. Increasing AS levels altered rumen papillae structure and surface coloration. The 6% AS group exhibited noticeably lighter mucosa with less dense papillae, while moderate AS levels (1.5–4.5%) showed more developed papillae and uniform coloration, consistent with the elevated VFA concentrations observed in these groups.

## 4 Discussion

This study demonstrated that AS supplementation produced dose-dependent effects on ruminal fermentation, trace mineral dynamics, metabolic responses, visceral development, and carcass-related traits in growing lambs. The findings indicate that AS primarily acted as a rumen-buffering agent capable of modifying ruminal physicochemical conditions and associated metabolic responses rather than directly enhancing growth performance. These observations are consistent with Abdelrahman, Ibrahim A. Alhidary (11) who previously reported that AS supplementation did not improve productive performance in lambs, suggesting that its main role is associated with rumen stabilization rather than growth promotion.

Moderate AS supplementation (1.5–4.5%) improved ruminal pH (5.7–5.9) and enhanced acetic and propionic acid concentrations, indicating improved fermentation efficiency. Maintenance of rumen pH within the physiological range is essential for sustaining normal fermentation activity and rumen epithelial function, particularly under high-concentrate feeding systems where endogenous salivary buffering may be insufficient. The improved rumen papillae appearance observed at moderate AS levels may therefore be associated with greater fermentation activity and increased VFA production. Similar responses have been reported for bicarbonate-based buffering systems that improved ruminal pH stability and fermentation characteristics in lambs and cattle (16, 31).

In contrast, excessive AS inclusion (6%) suppressed acetate and propionate concentrations despite maintaining relatively higher ruminal pH values. This finding suggests excessive buffering may have reduced overall fermentation intensity rather than improving fermentation efficiency. Previous studies have similarly reported that excessive dietary buffering can alter ruminal fermentation patterns and reduce microbial activity under highly buffered conditions (31, 32). Because rumen microbiota composition was not directly evaluated in the present study, interpretations regarding microbial responses remain speculative and are therefore limited to physiological indicators of fermentation.

Changes in ruminal trace mineral concentrations further reflected alterations in rumen conditions induced by AS supplementation. Ruminal fluid Cu, Zn, and Se concentrations increased progressively with increasing AS inclusion. These responses may be related to changes in ruminal pH and ionic environment affecting mineral solubility and dissociation. Moderate AS supplementation likely improved mineral availability through stabilization of ruminal conditions and enhanced fermentation activity. However, the marked elevations in Cu, Zn, and Se observed at 6% AS occurred simultaneously with reduced VFA concentrations, lighter rumen mucosa, and reduced papillae density, suggesting altered mineral redistribution under metabolically unfavorable conditions rather than improved mineral utilization. This is supported by strong inverse correlations between mineral levels and visceral and adipose tissue masses. Such a pattern aligns with established principles of mineral physiology, wherein oxidative or catabolic states can elevate circulating trace minerals through redistribution (28, 33).

The metabolic responses observed in the present study were consistent with the fermentation data. Increased serum NEFA concentrations and reduced glucose levels at higher AS inclusion rates suggest greater mobilization of body energy reserves. Because propionate serves as a major precursor for gluconeogenesis in ruminants, the reduction in propionic acid concentration at excessive AS levels may partially explain the lower circulating glucose concentrations. Concurrent reductions in visceral tissue weights and fat depots further support the occurrence of a mild catabolic state at high AS inclusion. In addition, the elevated ultimate meat pH observed in the 6% AS group may indicate reduced muscle glycogen availability before slaughter, which is commonly associated with impaired energy metabolism and physiological stress. These metabolic alterations are consistent with earlier reports showing that extreme rumen alkalinization reduces energy efficiency and tissue accretion (32).

Previous studies evaluating sodium bicarbonate and other buffering agents have reported inconsistent effects on ruminal fermentation and animal performance (34–36). Classical sodium bicarbonate supplementation typically increases rumen pH but has variable effects on VFA production (34); some studies report minimal changes (35), while others report improvements (36). The present findings suggest that the multi-ionic composition of AS may exert broader effects on ruminal physiochemistry than conventional single-component buffers. *In vitro* studies have also demonstrated that salivary constituents may influence fermentation characteristics beyond simple alkalinization (17). Salivary proteins and ions influence microbial activity beyond buffering (37, 38). Nevertheless, because direct microbial measurements were not conducted in the current study, the proposed mechanisms should be interpreted cautiously and require confirmation in future microbiome-based investigations.

The observed effects on visceral tissues and fat deposition are consistent with previous reports showing that buffering agents can influence nutrient utilization and carcass characteristics in lambs and cattle (14). Moderate AS supplementation maintained visceral tissue development and adipose reserves, whereas excessive supplementation reduced these parameters, suggesting impaired nutrient utilization under highly buffered conditions. Our identification of an optimal AS range of approximately 1.5–4.5% which align with Handayani, Sofyan (39) who refer to that the carbonate-based buffers are most effective at dietary levels of 0.7–1.5%. These findings support the concept that ruminal buffering may be beneficial only within an optimal inclusion range.

Overall, the present study indicates that moderate dietary supplementation of AS can improve ruminal fermentation characteristics and maintain metabolic stability in growing lambs. However, excessive supplementation negatively affected fermentation efficiency, metabolic responses, and carcass-related traits. The findings also suggest that ruminal Cu, Zn, and Se dynamics may reflect changes in ruminal physicochemical conditions under varying buffering levels. Further studies incorporating rumen microbiome analyses and nutrient digestibility measurements are needed to better elucidate the mechanisms underlying these responses and to validate the practical application of artificial saliva supplementation under commercial feeding conditions.

## 5 Conclusion

In conclusion, moderate supplementation of AS (1.5–4.5%) improved rumen fermentation, stabilized ruminal pH, and supported normal metabolic function and rumen tissue development in growing lambs. In contrast, excessive supplementation (6%) adversely affected fermentation efficiency, altered metabolic responses, and negatively influenced carcass characteristics. These findings indicate that AS may serve as a useful nutritional buffering strategy when included at moderate levels, whereas excessive inclusion should be avoided to prevent undesirable physiological and production-related effects.

## Data Availability

The original contributions presented in the study are included in the article/supplementary material, further inquiries can be directed to the corresponding authors.

## References

[1] Evci Ş. Digestion and importance of starch in ruminants. Turk J Vet Anim Sci. (2024) 8:143–50. doi: 10.47748/tjvr.1421153

[2] Banothu A Muskula AR Kotturi A. "Physiology of animal metabolism". In: Fundamentals of Veterinary Pathophysiology. Boca Raton, FL: CRC Press (2025). p. 48–55.

[3] Mahboubi A Holmström K Parchami M Uwineza C Agnihotri S Jomnonkhaow U . Volatile fatty acids in ruminants and their role as feed additives: a review. J Appl Anim Res. (2026) 54:2630934. doi: 10.1080/09712119.2026.2630934

[4] Zhu Z Zhang J Shah AM Zhang Q Bai B Hao L. Production, transport, and metabolism of volatile fatty acids in the yak rumen: Unraveling the unique mechanisms underpinning high-altitude adaptation. Microorganisms. (2026) 14:696. doi: 10.3390/microorganisms14030696, 41900455 PMC13028906

[5] Tuniyazi M Tang R Hu X Zhang N Shen P. Efficacy of carbonate buffer mixture in preventing hoof lamella injury associated with subacute ruminal acidosis in dairy goats. Vet Sci. (2024) 11:395. doi: 10.3390/vetsci11090395, 39330774 PMC11435902

[6] Udainiya S Tiwari A Ahirwar MK Mishra A. "Rumen Acidosis". In: Periparturient Diseases of Cattle (2024). p. 39–50.

[7] Mao J Wang L. Rumen acidosis in ruminants: a review of the effects of high-concentrate diets and the potential modulatory role of rumen foam. Front Vet Sci. (2025) 12:1595615. doi: 10.3389/fvets.2025.1595615, 40496917 PMC12148896

[8] Kumar BB Tariq H Mohanta RK Yaqoob MU Nampoothiri VM Mahesh M . "Rumen buffers to harness nutrition, health and productivity of ruminants". In: Feed Additives and Supplements for Ruminants. Berlin: Springer (2024). p. 495–518.

[9] Srivastava R Singh P Tiwari S Mishra D Kumar G. Sub-acute ruminal acidosis: understanding the pathophysiology and management with exogenous buffers. J Entomol Zool Stud. (2021) 9:593–9. doi: 10.22271/j.ento.2021.v9.i2i.8537

[10] Ungerfeld EM Cancino-Padilla N Vera-Aguilera N. "Fermentation in the rumen". In: Microbial Fermentations in Nature and as Designed Processes. Hoboken, NJ, Wiley: (2023). p. 133–65.

[11] Abdelrahman MM Alhidary IA Suliman GM Alobre MM Qaid MM . Physiological and productive impacts of including artificial saliva in lamb diets: growth, carcass traits, and fermentation efficiency. Vet Sci. (2026) 13:395. doi: 10.3390/vetsci13040395, 42076767 PMC13120013

[12] Harrison S. The Effects of Three Rumen Buffering Agents on Rumen Fermentation Parameters, Nutrient Digestibility, and Milk Composition in Dairy Cows. Pretoria: University of Pretoria (2021).

[13] Bilbilova EZ. Dietary Factors, Salivary Parameters, and Dental Caries. London: IntechOpen (2021).

[14] Alhidary I Abdelrahman M Elsabagh M. A comparative study of four rumen buffering agents on productive performance, rumen fermentation and meat quality in growing lambs fed a Total mixed ration. Animal. (2019) 13:2252–9. doi: 10.1017/S1751731119000296, 30819265

[15] Mahdavirad N Chaji M Bojarpour M Dehghanbanadaky M. Comparison of the effect of sodium bicarbonate, sodium sesquicarbonate, and zeolite as rumen buffers on apparent digestibility, growth performance, and rumen fermentation parameters of Arabi lambs. Trop Anim Health Prod. (2021) 53:465. doi: 10.1007/s11250-021-02909-7, 34546468

[16] Tripathi M Santra A Chaturvedi O Karim S. Effect of sodium bicarbonate supplementation on ruminal fluid Ph, feed intake, nutrient utilization and growth of lambs fed high concentrate diets. Anim Feed Sci Technol. (2004) 111:27–39. doi: 10.1016/j.anifeedsci.2003.07.004

[17] Palma-Hidalgo JM Belanche A Jiménez E Martín-García AI Newbold C Yáñez-Ruiz DR. Saliva and salivary components affect goat rumen fermentation in short-term batch incubations. Animal. (2021) 15:100267. doi: 10.1016/j.animal.2021.100267, 34102432

[18] Edwards JE Kim EJ Davies DR Hanafy R Kingston-Smith AH. Ruminant salivary microbes: passenger or player in the rumen? Microorganisms. (2023) 11:2390. doi: 10.3390/microorganisms11102390, 37894048 PMC10609091

[19] Wang B Li X Zhou Z Zhu Y Zuo Z Guo H. The role of five key minerals (cu, se, Zn, co, Fe) in reproductive function of female cattle: current insights and future directions. Vet Sci. (2026) 13:208. doi: 10.3390/vetsci13020208, 41746002 PMC12945088

[20] Kumar R Singh KD Chauhan SS Verma MK Verma AK Singh J . Trace minerals in growth, production and reproduction in farm animals. Indian J Anim Reprod. (2025) 46:1–12. doi: 10.48165/ijar.2025.46.01.1

[21] Katsoulos PD Bilgiç B Tarhan D Ateş F Ekin S Kozat S . Investigation of effects of low ruminal Ph values on serum concentrations of macrominerals, trace elements, and vitamins and oxidative status of dairy cows. Ruminants. (2025) 5:35. doi: 10.3390/ruminants5030035

[22] Nair PM Srivastava R Mani V Arulkumar S Tyagi N Mondal G. The importance of zinc, copper and manganese and their impact on growth, immunity and fertility of male cattle: a review. Biometals. (2025) 38:763–84. doi: 10.1007/s10534-025-00692-8, 40388044

[23] Vi RB McLeod K Klotz J Heitmann R. Rumen development, intestinal growth and hepatic metabolism in the pre-and postweaning ruminant. J Dairy Sci. (2004) 87:E55–65. doi: 10.3168/jds.S0022-0302(04)70061-2, 42143798

[24] Quille P Higgins T Neville EW Regan K O’Connell S. Evaluation and development of analytical procedures to assess buffering capacity of carbonate ruminant feed buffers. Animals. (2024) 14:2333. doi: 10.3390/ani14162333, 39199867 PMC11350906

[25] Sahib QS Ahmed HA Aafaq I Sheikh GG Ganai IA Ganai AM. Effect of feeding exogenous buffer (sodium bicarbonate) as feed additive on the performance of feedlot lambs. Indian J Anim Nutr. (2025) 42:32–9. doi: 10.56093/ijan.v42i1.5

[26] El-Katcha MI Soltan MAK Farfour HT El-Shobokshy SAS. Effect of different dietary buffer sources and roughage-to-concentrate ratios on growth performance, rumen fermentation, and health status of growing lambs. J Applied Vet Sci. (2025) 10:151–64. doi: 10.21608/javs.2025.390875.1633

[27] Asadi M Toghdory A Ghoorchi T Kargar S. The effects of diet concentrate and mineral buffer types on fattening lambs performance, nutrient digestibility, blood metabolites, rumen fermentation and carcass traits. Iranian J Applied Animal Sci. (2024) 14:1–32. doi: 10.3390/ani16040689

[28] Abdelrahman MM Swelum AA Ba-Awadh HA Al-Badwi MA Alobre MM Suliman GM . Ruminal solubility and bioavailability of trace minerals of growing lambs fed varying levels of live yeast with Total mixed ration. Front Vet Sci. (2025) 12:1657871. doi: 10.3389/fvets.2025.1657871, 41133191 PMC12542867

[29] Al-Ghamdi S Al-Baadani HH Alharthi AS Soufan W Suliman GM Abdelrahman MM . Influence of varied sprouted barley feeding levels on carcass traits, meat quality, and fatty acid profile of lambs. Cogent Food Agric. (2024) 10:2353669. doi: 10.1080/23311932.2024.2353669

[30] Alharthi AS Alshamiry FA Alghonaim AA Al-Baadani HH Alhidary IA. Relationship between forage particle size and rate of in vitro digestion parameters of alfalfa-based lamb in complete pelleted diets. Indian J Animal Res. (2025) 1:9. doi: 10.18805/IJAR.BF-1923

[31] Ramos SC Kim SH Jeong CD Mamuad LL Son A-R Kang SH . Increasing buffering capacity enhances rumen fermentation characteristics and alters rumen microbiota composition of high-concentrate fed Hanwoo steers. Sci Rep. (2022) 12:20739. doi: 10.1038/s41598-022-24777-3, 36456638 PMC9715728

[32] González L Ferret A Manteca X Calsamiglia S. Increasing sodium bicarbonate level in high-concentrate diets for heifers. I. Effects on intake, water consumption and ruminal fermentation. Animal. (2008) 2:705–12. doi: 10.1017/S1751731108001675, 22443595

[33] Goff JP. Invited review: mineral absorption mechanisms, mineral interactions that affect Acid–Base and antioxidant status, and diet considerations to improve mineral status. J Dairy Sci. (2018) 101:2763–813. doi: 10.3168/jds.2017-13112, 29397180

[34] Emmanuel B Lawlor M McAleese D. The effect of phosphate and carbonate-bicarbonate supplements on the rumen buffering Systems of Sheep. Br J Nutr. (2007) 24:653–60. doi: 10.1079/BJN19700066, 5470769

[35] Bilik K Strzetelski J Furgal-Dierzuk I Sliwinski B. Effect of supplementing Tmr diets with artificial saliva and acid Buf on optimizing ruminal Ph and fermentation activity in cows. Ann Anim Sci. (2014) 14:585–93. doi: 10.2478/aoas-2014-0043

[36] Mao S Huo W Liu J Zhang R Zhu W. In vitro effects of sodium bicarbonate buffer on rumen fermentation, levels of lipopolysaccharide and biogenic amine, and composition of rumen microbiota. J Sci Food Agric. (2017) 97:1276–85. doi: 10.1002/jsfa.7861, 27339112

[37] Ricci S Rivera-Chacon R Petri RM Sener-Aydemir A Sharma S Reisinger N . Supplementation with phytogenic compounds modulates salivation and salivary Physico-chemical composition in cattle fed a high-concentrate diet. Front Physiol. (2021) 12:645529. doi: 10.3389/fphys.2021.645529, 34149443 PMC8209472

[38] Zhang X Li Y Terranova M Ortmann S Kehraus S Gerspach C . A pilot investigation on the effect of induced saliva flow on digestive parameters in sheep, and a comparison with cattle. J Anim Physiol Anim Nutr. (2023) 107:1176–86. doi: 10.1111/jpn.13815, 36891877

[39] Handayani UF Sofyan A Sholikin MM Harahap MA Widodo S Bilyaro W . Preventing ruminal acidosis and optimizing ruminant performance by carbonate buffer supplementation: a review. Vet Integr Sci. (2026) 24:1–21. doi: 10.12982/Vis.2026.040

